# Diagnostic models for male lower urinary tract symptom management in primary care: a prospective, multicentre, diagnostic accuracy study

**DOI:** 10.3399/BJGP.2025.0513

**Published:** 2026-09-08

**Authors:** Chris Harding, Ridhi Agarwal, Janine Bates, Alison Bray, Sarah Milosevic, Emma Thomas-Jones, Michael Drinnan, Marcus Drake, Peter Michell, Bethan Pell, Haroon Ahmed, Natalie Joseph-Williams, Tom Schatzberger, Jane Davies, Kerenza Hood, Yemisi Takwoingi, Adrian Edwards

**Affiliations:** 1 Translational and Clinical Research Institute, Newcastle University, Newcastle-upon-Tyne, UK; 2 Freeman Hospital, Newcastle-upon-Tyne, UK; 3 Department of Applied Health Sciences, University of Birmingham, Birmingham, UK; 4 National Institute for Health and Care Research (NIHR) Birmingham, Biomedical Research Centre, University of Birmingham, Birmingham, UK; 5 Cardiff University, Cardiff, UK; 6 Northern Medical Physics and Clinical Engineering, The Newcastle upon Tyne Hospitals NHS Foundation Trust, Newcastle upon Tyne, UK; 7 Centre for Trials Research, Cardiff University, Cardiff, UK; 8 Teesside University, Middlesbrough, UK; 9 Department of Surgery & Cancer, Faculty of Medicine, Imperial College London, London, UK; 10 Newcastle Upon Tyne Hospitals NHS Trust, Newcastle Upon Tyne, UK; 11 DECIPHer, School of Social Sciences, Cardiff University, Cardiff, UK; 12 Division of Population Medicine, Cardiff University, Cardiff, UK; 13 CBC Health Federation, Gateshead, UK; 14 College of Biomedical & Life Sciences, Cardiff University, Cardiff, UK

**Keywords:** diagnosis, men's health, primary health care, urology

## Abstract

**Background:**

Recent research has concluded that primary care assessment and management of lower urinary tract symptoms (LUTS) requires practical resources.

**Aim:**

To develop and independently validate risk-prediction models for three common urological conditions based on a combination of simple, minimally invasive index tests in men with LUTS presenting to primary care.

**Design and setting:**

A prospective, multicentre, diagnostic accuracy study with independent development and validation cohorts set in 67 general practices in England and Wales.

**Method:**

A total of 601 men presenting to primary care with LUTS, 350 in the development cohort and 251 in the validation cohort, underwent index tests — validated symptom assessment, bladder diary, physical examination, serum prostate specific antigen, uroflowmetry, and post-void residual volume estimation. Invasive urodynamic studies provided a diagnostic reference standard. Risk-prediction models for urodynamic diagnoses were developed and then validated in independent cohorts. Discriminative and calibration performance of the risk-prediction models for diagnosing bladder outlet obstruction (BOO), detrusor underactivity (DU), and detrusor overactivity (DO) using the c-index, calibration slope and plot, and sensitivity and specificity of the models was undertaken.

**Results:**

The BOO model from the development cohort demonstrated good discriminative performance (optimism-corrected c-index of 0.80). The models derived from the development cohort for DU and DO demonstrated moderate discriminative ability (optimism-corrected c-indices of 0.64 and 0.67, respectively). Similar estimates of c-index were observed for each model within the independent validation cohort (BOO, 0.82; DU, 0.63; and DO, 0.62).

**Conclusion:**

Using a combination of simple, non-invasive index tests can accurately predict common urological diagnoses in men with LUTS, potentially facilitating earlier initiation of evidence-based treatments and improved management in primary care.

## How this fits in

Current assessment and management of lower urinary tract symptoms (LUTS) in primary care requires practical resources specifically around diagnostics. The aetiology of LUTS is varied and specifically in older men the results in the current study show that it should not be automatically assumed to be secondary to benign prostatic enlargement. The diagnostic decision tool detailed in this study has the potential to increase the level of accuracy surrounding the aetiology of lower urinary tract symptoms and hence guide primary care clinicians towards the most appropriate treatment.

## Introduction

Lower urinary tract symptoms (LUTS) in men are common, bothersome, and increasingly lead to secondary care referral. In the UK, LUTS affect one in four men, with around 300 000 per year consulting their GP. This represents a considerable long-term health burden.^1–3^ The results from a recent study reported a 7–8% secondary care referral rate for male LUTS. The authors concluded that ‘as provision for men with LUTS in primary care is inconsistent, primary care health professionals require practical resources to support the assessment of urinary symptoms’*.* ^4^

Current guidelines, including those from the National Institute of Health and Care Excellence (NICE),^5,6^ suggest that many men referred to specialist care with LUTS should be treated conservatively with lifestyle advice or simple medications, which should allow initial management to remain in primary care. After ruling out urinary tract infection and other serious causes of LUTS, the initial medical management of LUTS differs significantly according to underlying diagnosis. The reference standard diagnostic test for male patients with LUTS is urodynamic testing.^6^ This invasive test involves the measurement of bladder and abdominal pressures during both the filling and voiding phases of the micturition cycle. Urodynamics enable diagnoses for the more common causes of urinary symptoms, bladder outlet obstruction (BOO), detrusor underactivity (DU), and detrusor overactivity (DO), to be definitively made. However, urodynamic testing is invasive, requires expensive equipment, highly trained staff and currently takes place exclusively in secondary care.

With access to simple, accurate tests and clinical decision support tools, GPs could offer accessible, effective, timely management of LUTS in primary care. This would mirror the management of other long-term conditions such as diabetes, asthma, and hypertension. In the PRImary care Management of lower Urinary tract Symptoms in men (PriMUS) study^7^ the aim was to develop such a tool.

The objectives in this research were:

- to develop models to predict the likelihood of BOO, DU, and DO from simple non-invasive index tests that are feasible in primary care; and
- to estimate the diagnostic accuracy of these models in an independent validation cohort.

## Method

The study protocol has been previously published^7^ and more detailed information regarding the trial (trial registration: ISRCTN 10327305) can also be found in the NIHR Health Technology Assessment report.^8^ Public and patient involvement included design and management of the research, including developing participant information resources, analysing the research, contributing to the reporting, and dissemination of research findings.

In summary, men presenting to their GP with LUTS were recruited prospectively into two cohorts; one for development and one for validation of the diagnostic models. Participants underwent both simple index tests and the reference test (urodynamic studies) to determine which combination of index tests best predicted three common urodynamic diagnoses in men: BOO, DU, and DO. Interpretation of the urodynamic study was conducted by two independent reviewers providing internal validation of reference test results.

The index tests were symptom assessment using the International Prostate Symptom Score (IPSS) and International Consultation on Incontinence Questionnaire (ICIQ),^9,10^ completion of a bladder diary, physical examination including digital rectal examination, serum prostate specific antigen, home and in-clinic uroflowmetry, and post-void residual volume estimation with ultrasound. Patients then underwent invasive urodynamic studies according to a standardised operating procedure to provide a diagnostic reference.

Sample size calculations were performed separately for the model development and validation cohorts. For both, estimated prevalences for BOO, DU, and DO of 31%, 16% and 57% were used, respectively, based on previous literature^11,12^ and clinical expertise.

The sample size for developing the risk-prediction models was based on a rule of thumb suggesting that five events per variable was adequate.^13^ A sample size of 350 was chosen to allow at least 11 variables in each model. This was driven by the lowest estimated prevalence of 16% for DU, giving 56 events (diagnoses). Over 100 events were expected for the BOO model and 200 for the DO model providing a more than adequate number.

The required sample size for the validation cohort was chosen to ensure that estimates of test accuracy could be made with adequate precision. Sensitivity and specificity of 75% were deemed to be the minimum clinically useful performance. A sample size of 325 was chosen, giving estimates of sensitivity of 75% to within 8%, 10% and 14% for DO, BOO, and DU, respectively, based on ‘positive’ samples of 185, 101, and 52, and estimates of specificity of 75% to within 8%, 7%, and 6%, based on ‘negative’ samples of 140, 224, and 273. To allow an attrition rate of 20–25%, the resulting 675 was increased to give a final required sample size of 880.

Sensitivity analyses were performed by fitting two alternative models for each target condition. In sensitivity analysis 1 (SA1), predictors that may be difficult to obtain in practice were excluded from the list of candidate predictors (mean urgency score and mean 24-hr fluid intake from the bladder diary). In sensitivity analysis 2 (SA2), alternative measures or methods of measurement of candidate predictors were considered (for example, using measurements from in-clinic flow tests rather than home uroflowmetry).

Risk-prediction models were developed for each target condition using logistic regression. Multiple imputation by chained equations was used to handle missing data and fractional polynomial functions were used to fit continuous variables. Discrimination (ability to distinguish between those who do or do not have the target condition) of each model was assessed using the c-index, and calibration (agreement between predicted and observed probabilities) was assessed using calibration plots and calibration slope. Internal validation was conducted to assess optimism of the performance statistics using bootstrapping. External validation was conducted to assess model performance in another sample from a similar population as the development cohort (validation cohort). In both forms of validation, the models were recalibrated using the calibration slope as a shrinkage factor to re-estimate the intercept and model coefficients. Sensitivity and specificity were plotted on a receiver operating characteristic plot for each model. Risk thresholds were identified at a sensitivity and specificity of 75%, which was deemed to be the minimum clinically useful performance.

## Results

There were 350 and 251 men recruited into the development and validation cohorts, respectively. The recruitment for the validation cohort was less than planned, largely as a result of the COVID-19 restrictions imposed during the trial period. Baseline characteristics of men recruited to development and validation cohorts are shown in Table 1. In the development cohort (N = 350), the median age was 69.0 years (interquartile range 61.8–74.9 years) and the majority (*n* = 341, 97.4%) of the men were of white ethnicity. Almost half (*n* = 171/350, 48.9%) of the men were ex-smokers, 150/350 (42.9%) had never smoked and 29/350 (8.3%) were current smokers. Over two-thirds of participants had comorbidities: 107/350 (30.6%) had no comorbidities, 188/350 (53.7%) had a single comorbidity, and the remaining 55/350 (15.7%) had multiple comorbidities. There were 41/350 (11.7%) men with diabetes mellitus II, 13/350 (3.7%) with obstructive sleep apnoea, and 12/350 (3.4%) with cerebrovascular accident. There were 102/350 (29.1%) men on alpha-blockers, 65/350 (18.6%) on calcium channel blockers, 26/350 (7.4%) on selective serotonin reuptake inhibitors antidepressants, and 25/350 (7.1%) participants on 5-alpha reductase inhibitors. Generally, the baseline characteristics of participants in the validation cohort (N = 251) were similar to those of the development cohort. The validation cohort consisted of more never smokers (132/251, 52.6%) and fewer ex-smokers (98/251, 39.0%) than the development cohort.

Summary statistics of the candidate predictors are shown in Table 2.

**Table 1.** Participant demographics and clinical characteristics of the development and validation cohorts

**Characteristic**	**Development cohort (** *N* = 350)	**Validation cohort (** ***N*** **= 251)**
**Prevalence**		
BOO	163 (46.6)	112 (44.6)
DU	141 (40.3)	87 (34.7)
DO	253 (72.3)	166 (66.1)
**Age, years**		
Median (IQR)	69.0 (61.8−74.9)	67.6 (60.6−74.0)
Missing	0 (0.0)	0 (0.0)
Height, cm		
Median (IQR)	176.0 (171.0−180.0)	176.0 (172.0−181.0)
Missing	1 (0.3)	5 (2.0)
**Weight, kg**		
Median (IQR)	83.5 (75.0−96.0)	86.0 (76.7−97.5)
Missing	0 (0.0)	7 (2.8)
**Ethnicity**		
White	341 (97.4)	237 (94.4)
Asian (Pakistani)	1 (0.3)	2 (0.8)
Asian (Chinese)	1 (0.3)	0 (0.0)
Asian (Indian)	2 (0.6)	3 (1.2)
Asian (Other)	2 (0.6)	1 (0.4)
Black (Caribbean)	2 (0.6)	1 (0.4)
Black (African)	0 (0.0)	2 (0.8)
Black (Other)	0 (0.0)	1 (0.4)
Other	1 (0.3)	2 (0.8)
Missing	0 (0.0)	2 (0.8)
**Smoking status**		
Never	150 (42.9)	132 (52.6)
Current	29 (8.3)	17 (6.8)
Ex-smoker	171 (48.9)	98 (39.0)
Missing	0 (0.0)	4 (1.6)
**Medical history**		
None	107 (30.6)	70 (27.9)
Adrenal insufficiency	0 (0.0)	0 (0.0)
Cerebrovascular accident	12 (3.4)	17 (6.8)
Chronic heart failure	2 (0.6)	6 (2.4)
Chronic venous stasis	0 (0.0)	2 (0.8)
Dependent oedema	3 (0.9)	2 (0.8)
Diabetes insipidus	0 (0.0)	0 (0.0)
Diabetes mellitus I	1 (0.3)	0 (0.0)
Diabetes mellitus II	41 (11.7)	28 (11.2)
Hypercalcaemia	3 (0.9)	0 (0.0)
Liver failure	0 (0.0)	0 (0.0)
Obstructive sleep apnoea	13 (3.7)	12 (4.8)
Polyuric renal failure	0 (0.0)	0 (0.0)
Pyelonephritis	0 (0.0)	0 (0.0)
Sickle cell anaemia	1 (0.3)	0 (0.0)
Other	232 (66.3)	167 (66.5)

Data are *n* (%) unless otherwise specified. BOO = bladder outlet obstruction. DO = detrusor overactivity. DU = detrusor underactivity. IQR = interquartile range.

**Table 2.** Summary of candidate predictors by the development and validation cohorts

Candidate predictor and analysis	Development cohort (*N* = 350)	Validation cohort (*N* = 251)
**Age, years (participant demographics)** ^**a,b,c**^		
Median (IQR)	69.0 (61.8–74.9)	67.6 (60.6-74.0)
Missing, *n* (%)	0 (0.0)	0 (0.0)
**Size of prostate (digital rectal examination),** ^**a,b,c**^ ***n* (%)**		
Normal	101 (28.9)	68 (27.1)
Mild enlargement	101 (28.9)	79 (31.5)
Moderate enlargement	137 (39.1)	95 (37.8)
Gross enlargement	11 (3.1)	7 (2.8)
Missing	0 (0.0)	2 (0.8)
**Prostate specific antigen test, ng/mL (blood test)** ^**a,b,c**^		
Median (IQR)	1.6 (0.9–2.8)	1.3 (0.7–2.5)
Missing, *n* (%)	0 (0.0)	2 (0.8)
**Incontinence symptoms subscore (ICIQ-MLUTS)** ^**a,b**^		
Median (IQR)	4.0 (3.0–7.0)	4.0 (3.0–7.0)
Missing, *n* (%)	0 (0.0)	0 (0.0)
**Voiding symptoms subscore (ICIQ-MLUTS)** ^**a,b**^		
Median (IQR)	7.5 (5.0–10.0)	7.0 (4.0-10.0)
Missing, *n* (%)	0 (0.0)	0 (0.0)
**Post-void residual urine, mL (bladder ultrasound)** ^**a,b**^		
Median (IQR)	67.0 (29.0–150.0)	48.0 (18–114)
Missing, *n* (%)	9 (2.6)	8 (3.2)
**Median maximum flow rate, mL/s (home uroflowmetry)** ^**a,b**^		
Median (IQR)	12.1 (9.1–16.6)	12.7 (9.2–17.2)
Missing, *n* (%)	0 (0.0)	39 (15.5)
**Median voided volume, mL (home uroflowmetry)** ^**a,b**^		
Median (IQR)	180.0 (139.0–233.0)	183.0 (137–254)
Missing, *n* (%)	0 (0.0)	40 (15.9)
**Mean 24-h frequency (home uroflowmetry)** ^**a,b**^		
Median (IQR)	7.4 (5.6–9.2)	7.8 (5.9–10.1)
Missing, *n* (%)	0 (0.0)	40 (15.9)
**Mean nocturia frequency (home uroflowmetry)** ^**a,b**^		
Median (IQR)	1.6 (1.0–2.4)	1.8 (1.2–2.8)
Missing, *n* (%)	4 (1.1)	41 (16.3)
**Mean urgency score (bladder diary)** ^**a,c**^		
Median (IQR)	1.5 (1.1–2.1)	1.6 (1.0–2.3)
Missing, *n* (%)	20 (5.7)	44 (17.5)
**Mean 24-h fluid intake, mL (bladder diary)** ^**a,c**^		
Median (IQR)	1830.0 (1412.0–2236.5)	1833.0 (1433.0–2340.0)
Missing, *n* (%)	34 (9.7)	60 (23.9)
**Incontinence symptoms subscore (IPSS questionnaire)** ^**c**^		
Median (IQR)	9.0 (6.0–11.0)	9.0 (6.0–11.0)
Missing, *n* (%)	0 (0.0)	0 (0.0)
**Voiding symptoms subcore (IPSS questionnaire)** ^**c**^		
Median (IQR)	7.0 (4.0–11.0)	7.0 (3.0–11.0)
Missing, *n* (%)	2 (0.6)	0 (0.0)
**Palpable bladder (physical examination),^c^ *n* (%)**		
Yes	11 (3.1)	8 (3.2)
No	339 (96.9)	241 (96.0)
Missing	0 (0.0)	2 (0.8)
**Maximum flow rate, mL/s (in-clinic flow test)** ^**c**^		
Median (IQR)	7.6 (4.0–11.1)	7.7 (5.0–13.0)
Missing, *n* (%)	36 (10.3)	23 (9.2)
**Voided volume, mL (in-clinic flow test)** ^**c**^		
Median (IQR)	114.0 (58.0–238.0)	123.5 (70.0–244.0)
Missing, *n* (%)	29 (8.3)	21 (8.4)
**Average number of voids (bladder diary)** ^**c**^		
Median (IQR)	9.3 (7.3–11.3)	9.0 (7.3–11.0)
Missing, *n* (%)	34 (9.7)	59 (23.5)
**Nocturnal polyuria — proportion of 24-h urine output produced during sleeping hours (bladder diary)** ^**c**^		
Median (IQR)	0.3 (0.3–0.4)	0.3 (0.2–0.4)
Missing, *n* (%)	48 (13.7)	74 (29.5)

^a^Included in main analysis; ^b^Included in sensitivity analysis 1; ^c^Included in sensitivity analysis 2. ICIQ-MLUTS = International Consultation on Incontinence Questionnaire — Male Lower Urinary Tract Symptoms. IPSS = International Prostate Symptom Score. IQR = interquartile range.

Of 601 men recruited, 545 (90.7%) had ≥1 of the three common urological conditions on urodynamic testing, with prevalence from 34.7% (DU in validation cohort, 87/251) to 72.3% (DO in development cohort, 253/350).

Models were developed and validated for BOO, DU, and DO (Table 3). Six index tests were required for the three models (age, prostate specific antigen test, voiding symptoms subscore [ICIQ — MLUTS], median maximum flow rate, median voided volume and post-void residual urine). Optimism-corrected calibration slopes <1 suggest that all models were overfitted at development, although the BOO model demonstrated good calibration performance at validation (Table 3).

**Table 3.** Performance measures of the risk-prediction models in the main analysis

	Development cohort		Validation cohort			
	Optimism- corrected calibration slope	Optimism-corrected c-index	Calibration slope (95% CI)	C-index (95% CI)	Sensitivity at 75% specificity (threshold)	Specificity at 75% sensitivity (threshold)
**BOO**	0.87	0.80	0.99 (0.68 to 1.30)	0.82 (0.76 to 0.88)	71.3% (0.533)	74.2% (0.509)
**DU**	0.77	0.64	0.82 (0.31 to 1.32)	0.63 (0.56 to 0.71)	39.8% (0.414)	47.3% (0.342)
**DO**	0.78	0.67	0.72 (0.27, 1.17)	0.62 (0.55, 0.70)	33.3% (0.752)	45.6% (0.638)

BOO = bladder outlet obstruction. DO = detrusor overactivity. DU = detrusor underactivity. CI = confidence interval.

In the development cohort, the BOO model demonstrated good discriminative performance with an optimism-corrected c-index of 0.80, whereas the models for DU and DO were moderately discriminative (Table 3). This was evident in the diagnostic performance of the validation cohort where only the BOO model approached the criterion of >75% sensitivity and specificity.

### Sensitivity analysis

The diagnostic performance of the risk-prediction models for BOO, DU, and DO from SA1 remained the same with the exclusion of the mean urgency score and the mean 24-hr fluid intake, suggesting that the models were robust and the exclusion of these parameters did not affect the model’s performance. A total of six index tests were required for the three models in SA2 (age, prostate specific antigen test, maximum flow rate, voided volume, mean 24-h fluid intake, and storage symptoms subscore [IPSS]). The optimism-corrected c-index reduced significantly for the BOO model (0.69), but was comparable for DU (0.63), and DO (0.65). The optimism-corrected calibration slope was <1 for all models, suggesting overfitting (BOO, 0.83; DU, 0.80; DO, 0.74).

## Discussion

### Summary

The PriMUS study shows that in a cohort of men presenting to primary care with LUTS, a combination of non-invasive clinical assessments can be used to estimate the likelihood of common urological conditions with a degree of accuracy comparable with existing primary care clinical support tools in other disease areas.^14^

### Strengths and limitations

The PriMUS study was conducted in line with current Good Clinical Practice and topic-specific research guidelines^14^ and oversight throughout was provided by an external trial steering committee. In this study urodynamic testing was used as the reference standard for diagnosis and the interpretation of this test was conducted by two independent reviewers providing internal validation of the reference test results.

The sample size of 601 men recruited from different geographical, socioeconomic, and ethnic backgrounds makes this one of the largest studies in this field. The sample was lower than protocol targets mainly because urodynamic studies necessitate regular coughs for quality-control purposes and there was reticence regarding clinic attendance during COVID. The prevalence of the three diagnoses was considerably higher than the authors of the current study had assumed for the sample size calculation and therefore this lower sample size did not increase the width of the confidence intervals to a level that would reduce the authors’ ability to draw robust conclusions.

A limitation of the study is that in terms of diagnostic accuracy the model only approached the pre-specified sensitivity and specificity threshold for clinically useful test performance for the diagnosis of BOO. The authors of the current study believe that this alone will be useful for initial LUTS assessment in men presenting to primary care. The majority of LUTS presentations are likely to be from older men and the logical assumption is that the underlying diagnosis would be BOO owing to benign prostatic enlargement. However, the current data would refute that and in the current cohort of men with a median age of >68 years the most prevalent diagnosis was DO. Hence the ability to accurately predict the presence or absence of BOO would potentially avoid inappropriate first-line empirical treatment directed at a possible BOO diagnosis that may not be present.

Another limitation of the study is that it does not evaluate the possible impact of pathway change for men presenting with LUTS, specifically with regard to outcomes important to patients and potential cost-savings for the healthcare system. The potential for primary care clinicians to accurately diagnose and initially manage these patients is, however, demonstrated by the current results.

Finally, the authors acknowledge there is large uncertainty around the calibration slope, likely driven by the smaller than planned sample size of the validation cohort during the COVID-19 pandemic. Thus, further external validation and recalibration may be required before the models can be widely used in practice. This may ensure applicability of the models in settings where case mix or prevalence could differ to ensure the calibration performance of the models. It is also acknowledged that the ‘five events per variable’ rule is now outdated, as this often leads to insufficient sample size, especially for complex models, which in turn can result in the models overfitting. However, at the time of study development this rule was accepted.

### Comparison with existing literature

Various simple tests have been previously assessed for diagnostic accuracy, most often regarding their ability to predict BOO. Symptom assessment alone was associated with poor diagnostic accuracy in two large multinational studies.^15,16^ Maximum urine flow rate, a widely used variable obtained from a non-invasive urine flow study, had no correlation with urinary symptoms and was unable to accurately diagnose urological conditions such as BOO^17^. Similarly, post-void residual volume was found to have a weak correlation with BOO.^18,19^ However, the diagnostic ability of serum prostate specific antigen (PSA) was found to have a significant correlation with BOO.^20^ Despite this the current use of serum PSA measurement is largely confined to its ability to predict underlying prostate cancer.

The existing literature suggests that when used alone, simple tests do not carry sufficient levels of accuracy to be useful in the diagnosis of male LUTS. Other researchers have investigated the use of a combination of measures derived from simple tests to provide a urodynamic diagnosis of BOO.^21^ A combination of clinical measurements including prostate volume, maximum flow rate, and post-void residual volume was shown to better predict BOO than symptom scores alone. These findings align with the results in the current study. The PriMUS study used a larger cohort and more robust methodology than previously published studies and showed that combining results from simple tests can accurately diagnose the cause of male LUTS. More recently, the UPSTREAM study identified that core evaluations used in primary care may predict outcomes of interventional treatment^22,23^ further suggesting that simple, non-invasive tests have significant clinical utility.

Another important finding from the PriMUS study is the high prevalence of defined urodynamic abnormalities in men presenting to primary care with LUTS. The current findings are supported by other data that estimate a high prevalence of underlying urodynamic abnormalities but in an unselected population.^24^

### Implications for research and practice

Further research to evaluate the use of the PriMUS diagnostic tool in practice, particularly in terms of potential effects on patient-important outcomes and resource use (practitioner time, medications, referrals), is necessary before full integration into clinical pathways. Both clinical effectiveness and process evaluations will provide valuable information for wider implementation and examine which patients may most benefit from increased use of simple diagnostic tests in primary care, such as those who may have failed empirical first-line treatment.

Previously published qualitative work from PriMUS indicates that patients prefer initial LUTS management in primary care.^25^ These findings mirror the views expressed in a 2024 report that revealed that the public in the UK wants primary and community care to be a higher priority for NHS resources than hospital services.^26^

Despite the high prevalence of urological conditions identified in this study, baseline data from participants indicated that most men had not yet tried potentially suitable first-line medical treatments. The PriMUS clinical decision support tool, using these diagnostic models and with its management algorithm based on existing NICE guidelines, could facilitate the initial accurate management of these patients in a primary care setting while also avoiding a ‘trial and error’ approach of different medications.^8^ For example, most symptomatic men in the current study were >60 years old and an underlying diagnosis of BOO might have been assumed. In both cohorts <50% were found to actually have BOO indicating that a trial of alpha-adrenoreceptor antagonists, which would be common practice in this scenario, would not be successful. A different approach based on simple, rapidly available diagnostic tests such as illustrated in the PRiMUS study is likely to result in those men getting the most appropriate treatment more quickly and avoiding repeat consultations, thus potentially saving costs.

The levels of diagnostic accuracy reported in the current study are comparable with other (non-urological) predictive models that are routinely used in primary care, such as QRisk,^27^ HAS-BLED^28^ and CHADS-VASC2^29^ albeit with different timespans for predicting risk. These other scales/scores are used routinely and integrated into clinical record systems in primary care. This provides some confidence that with implementation in routine settings there will be opportunity for the PriMUS risk- prediction models to be made available via the standard primary care record systems (EMIS, Vision, SystemOne).

The variables included in the PriMUS study's proposed model and clinical diagnostic tool include simple demographic data, symptom scores, and PSA tests, already routinely administered by primary care staff. Other variables required for the model include simple measurements that can be obtained from a home uroflowmetry test. This study found 92.5% (556/601) of men successfully used the home uroflowmetry device and provided usable data to input into the model. This suggests that the addition of a simple home uroflowmetry test, used alongside existing investigations carried out in primary care, may enable men presenting with LUTS to be diagnosed and potentially treated within general practice. Another predictive variable (for DO and DU) that does present more challenges is post-void residual volume estimation (from ultrasound). The authors of the current study envisage implementation via a pathway involving a GP with special interest, operating at the level of a cluster or network of practices or community diagnostic clinics. Primary care capacity to undertake such clinical management requires resourcing but could be funded from costs potentially saved by avoiding early secondary care referral.

In conclusion, the PriMUS study has shown the potential for combining the results of simple index tests to improve the diagnosis of the three common causes of LUTS in men in primary care. The high prevalence of urodynamic abnormalities within a cohort of men presenting to primary care with LUTS emphasises the clinical need for better and more widespread access to diagnostics and initial treatment, and suggests that this could take place within primary care, aligning with patient preferences.

## Data

Datasets available from the authors on request.
